# Plausible Impact of Dietary Habits on Reduced Blood Sugar in Diabetic Opium Addicts with Coronary Artery Disease

**Published:** 2012-09-15

**Authors:** Mahdi Najafi, Mehrdad Sheikhvatan

**Affiliations:** 1 Anesthesiology Department, Tehran Heart Center, Tehran University of Medical Sciences, Tehran, IR Iran; 2 Research Department, Tehran Heart Center, Tehran University of Medical Sciences, Tehran, IR Iran

**Keywords:** Opium, Nutrition, Diabetes Mellitus, Coronary Artery Disease

## Abstract

**Background:**

The effect of opium on glycemic control in diabetics is a controversial issue, as some studies have shown glucose lowering effect of opium in diabetes while the results of other studies do not support this idea. The possible role of opioid peptides in the regulation of food intake has been previously investigated. However, there is no data available about relationship between opium using and dietary pattern.

**Objectives:**

The aim of the present study was to determine the daily intake of different nutrients in opium addict with diabetes diagnosed with coronary artery disease (CAD).

**Methods:**

This study comprised 232 consecutive diabetic patients with CAD, and candidates for isolated coronary artery bypass surgery in Tehran Heart Center. Of these, 26 patients were opium addicts. Nutritional assessment was obtained by a validated semi-quantitative food frequency questionnaire (FFQ).

**Results:**

In opium addicts compared to non-addicts, consumption of carbohydrates (360.0±120.9 versus 447.8±249.8 Gr/day, P=0.016) and vitamin A (1170.4±570.2 versus 1496.3±889.6 μg/d as Retinol Activity Equivalent (RAE), P=0.040) was lower than non-addicts and intake of other nutrients were similar across two group of patients.

**Conclusions:**

Opium addiction in diabetic patients may lead to decrease of vitamin A and carbohydrate intake. This study showed that carbohydrate intake in addicted diabetic patients is lower than their non-addict counterpart. Thus, the so called lowering effect of opium on blood sugar may be due to nutritional habit of addicted patients.

## Introduction

The impact of drug addiction on metabolic systems, especially in diabetic patients has been reported previously. It is known that opium smoking could lead to serum glucose and high density lipoprotein alterations and thus worsens metabolic disorders in diabetics (1). It has also been found that drug addiction could cause dysfunctional eating patterns. Behavioral interaction between food and substance abuse has already been demonstrated (2). Furthermore, drug addicts without organic pathology were undernourished so that in a study by Santolaria-Fernández et al. more than 90% of them weighed under the mean weight for the population and more than half had a weight loss above 5% (3). In addition, opium plays a pivotal role as a risk factor for coronary artery disease especially in diabetics and the amount of opium consumed was significantly associated with the severity of coronary atherosclerosis in these patients (4).

Thus it is evident that both diabetes and opium addiction are risk factors for CAD which is under influence of dietary pattern. But it is not clear if dietary pattern is different in addict and non-addict diabetics. A question prevailed among diabetic patients is whether opium decreases blood sugar. In this context, data are controversial (1,5,6). Although a few studies have reported relationship between food intake and substance abuse (2,3), there is no such data available about dietary pattern in opium addicts. Whether low blood sugar in opium addicts with diabetes reported in some studies (5,6) related to their different pattern of nutrition was the subject of present investigation, where we aimed to evaluate the daily dietary intake in diabetic patients with and without addiction to opium.

## Materials and Methods

This study comprised 232 consecutive diabetic patients diagnosed with coronary artery disease and candidates for isolated coronary artery bypass surgery in Tehran Heart Center. Of these, 26 patients were opium addicts. The data considered for analysis included demographic characteristics, preoperative risk factors, paraclinical data, and cardiac status. Opium dependence was defined according to the DSM-IV criteria for substance dependence (7) considering regular daily consumption. The patients were also interviewed on admission to surgical ward and before CABG operation and asked to report how often they consumed each of the food items listed as the number of times per day, per month or per year during previous year. Nutritional assessment was obtained by a validated semi-quantitative food frequency questionnaire (FFQ), previously validated in IR Iran (8) and a 24-hour dietary recall questionnaire to record the types, amounts and frequencies of food consumed. We used the sum of the consumption of each of several food items to estimate the overall consumption of the food group to which each item belonged. Details of our methods are discussed in another report (9). Informed consent was obtained from all patients after briefing them about the study which was approved by the Ethic’s Committees of Tehran University of medical sciences.

Results were reported as mean ± standard deviation (SD) for quantitative variables and percentages for categorical variables. Categorized variables were compared using chi-square test, or Fisher’s exact test if required. Student’s t-test was used to compare the means of food items between addict and non-addict groups. P values of 0.05 or less were considered statistically significant. The statistical analyses were performed using SPSS version 13.0 for windows (SPSS Inc., Chicago, IL, USA).

## Results

Demographic characteristics and clinical data of patients are summarized in Table 1. Body mass index was significantly lower in opium addict patients. Education level was similar in both groups. Among risk factors for coronary artery disease, recent myocardial infarction and renal failure were more prevalent among addicts. Also, addict patients consumed more alcohol than non-addicts. The mean of ejection fraction was lower in addicts than non-addict patients, while function class and numbers of coronary arteries involvement were similar in both groups. Among laboratory indicators, latest creatinine level was higher in addict group and other indices including lipid profile were similar in both. Though last fasting blood sugar was lower in addict patients, the difference was not statistically significant.

**Table 1:** Demographic Characteristics and Clinical Data of Studied Patients

Characteristics		Opium addicts(n=26)	Non-addicts(n=206)	P value
**Men (%)**		96.2	58.3	<0.001
**Age (year)**		57.0±7.3	59.3±8.4	0.145
**Body mass index (kg/m^2^)**		26.9±3.2	28.5±4.3	0.025
**Education level**	**Primary (%)**	50.0	60.2	0.592
	**Secondary (%)**	30.8	25.7	
	**University (%)**	19.2	14.1	
**Family history of CAD‡ (%)**		57.7	49.5	0.432
**Current cigarette smoking (%)**		73.1	24.3	<0.001
**Alcohol using (%)**		28.0	9.2	0.012
**Hypercholesterolemia (%)**		61.5	78.2	0.060
**Renal failure (%)**		23.1	8.7	0.036
**Hypertension (%)**		53.8	59.2	0.600
**Cerebrovascular disease (%)**		3.8	6.3	0.999
**Peripheral vascular disease (%)**		26.9	39.3	0.220
**Recent myocardial infarction (%)**		72.0	42.9	0.006
**Functional class**	**I (%)**	38.5	32.5	0.146
	**II (%)**	57.7	48.1	
	**III (%)**	3.8	19.4	
**Coronary vesselsinvolvement**	**Single-vessel disease (%)**	3.8	2.4	0.612
	**Two-vessel disease (%)**	11.5	18.9	
	**Three-vessel disease (%)**	84.6	78.6	
**Ejection fraction (%)**		45.0±9.5	49.9±10.0	0.019
**Euroscore**		1.9±2.1	2.5±2.3	0.175
**Last fasting blood sugar (mg/dl)**		118.9±2.5	127.3±46.3	0.349
**Last creatinine (mg/dl)**		1.4±0.5	1.2±0.3	0.002
**Triglyceride (mg/dl)**		165.3±74.7	177.0±79.9	0.464
**Cholesterol (mg/dl)**		146.7±33.6	160.2±49.1	0.075
**High density lipoprotein (mg/dl)**		38.5±8.0	40.3±8.6	0.287
**Low density lipoprotein (mg/dl)**		77.5±29.9	84.3±35.5	0.291
**Hemoglobin A_1_C (%)**		6.8±1.7	6.9±1.6	0.879
**Albumin (g/dl)**		4.6±0.4	4.6±0.3	0.861

As shown in Table 2, the daily consumption of carbohydrates and vitamin A in opium addicts was less than non-addicts. With respect to the intake of other nutrients, there were no statistically significant differences between the two groups.

**Table 2:** Daily Values of Nutrient Intake in Opium Addicts and Non-Addict Patients

Nutrients intakes	Opium addicted (n=26)	Non-addicted (n=206)	P value
**Energy (Kcal/d)**	2478.1±824.6	2909.3±1397.3	0.063
**Carbohydrate (gr/d)**	360.0±120.9	447.8±249.8	0.016
**Protein (gr/d)**	90.8±29.6	104.2±45.0	0.099
**Total fat (gr/d)**	81.0±36.8	85.7±42.3	0.621
**Saturated fat (gr/d)**	31.4±14.6	32.9±17.8	0.697
**Monounsaturated fat (gr/d)**	31.2±15.6	32.5±16.2	0.744
**Polyunsaturated fat (gr/d)**	17.7±9.0	20.1±11.6	0.308
**Calcium (mg/d)**	1151.6±430.6	1306.8±471.1	0.165
**Folate (mg/d)**	452.0±146.0	511.7±206.9	0.129
**Vitamin B12 (mg/d)**	5.4±2.6	5.9±3.6	0.510
**Vitamin E (mg/d)**	9.5±2.5	10.7±4.3	0.081
**Vitamin A (μg/d as RAE)**	1170.4±570.2	1496.3±889.6	0.040

To rule out the possible confounding effect of cigarette smoking, we compared the daily intake of both carbohydrate and vitamin A between cigarette smokers and non-smokers in diabetics. Univariate analysis showed no significant difference in daily intake of carbohydrate (432.4±262.7 Gr/day in smokers and 442.0±232.8 Gr/day in non-smokers; P=0.816) and vitamin A (1415.0±817.8 µg/d as Retinol Activity Equivalent (RAE) in smokers and 1484.7±890.1 µg/d as RAE in non-smokers; P=0.610). Therefore, current cigarette smoking did not influence the intakes of these two types of dietary components in the subjects under study.

## Discussion

In the present study, we found that the consumption of carbohydrate in addict patients with diabetes mellitus was significantly lower than non-addicts. The possible role of opioid peptides in the regulation of food intake has previously been investigated (10). Some studies show that opioid receptor agonists can suppress the intake of carbohydrate diet (11,12). However there is no such study in opium addict patients.

It is a common belief among addict patients that opium consumption decreases the level of blood glucose thus resulting in a better management of diabetes. As opium smoking is predominant form of substance abuse in IR Iran (13) and most common in patients with CAD (14), it was deemed necessary to study the effect of opium addiction on blood sugar level especially in patients with diabetes.

According to our results ,there was no significant difference found between two groups of addict and non-addict diabetics with regard to indices of short-term and long -term glycemic control including fasting blood sugar (FBS) and glycated hemoglobin or HbA1c (Table 1). FBS in addict patients was slightly lower than non- addicts without any significant difference which may be due to the number of addicts in our study.

There are conflicting reports regarding blood sugar level in opium addiction (1,5,6,15). Thus, in our opinion there are other factors to be considered in order to conclude this controversy, and most important of them is the low carbohydrate intake. Our findings of no significant difference between FBS level in addicts and non-addicts may be explained by stressful condition imposed by existing CAD and proposed coronary artery bypass that offset the effect of low carbohydrate intake on blood sugar level in addicts. CAD impact on addict patients may be more pronounced than non-addicts because of their sensitivity and stronger response to noxious stimulations. Studies on the relationship of opium to blood glucose are not reported in connection with CAD patients. (1, 5, 6, 15)

Although we found adverse correlations between daily intake of carbohydrate and two known determinants of outcome i.e. serum creatinine concentration (β=-143.3, P=0.029) and ejection fraction (β=-3.8, P=0.040), no relationship was found between dietary carbohydrate and renal and cardiac function indices. The clinical importance of these two findings warrants further investigation.

Among nutrients, intake of vitamin A in opium-addict patients was significantly lower than non addicts. It has been previously found that vitamin malnutrition, as judged from circulating levels, was common among addict population (16). Probable reasons of this malnutrition are due to the low consumption of fruit and vegetables as main sources of vitamins in drug addicts compared to general population as they are more inclined to consume food low in vitamin content. Therefore, the opium addicts may have vitamin deficiencies, especially antioxidant vitamins (17). In addition, a partial reduction in vitamin levels may be probably due to cigarette smoking (18). In our study, the majority of addict patients smoked cigarette and history of cigarette smoking was significantly more frequent in addicts. In a study by Nazrul Islam et al. it was indicated that retinol in the drug addicts were significantly low as compared to those in the cohort controls and this reduction was more noticeable among multiple drug addicts (19). Moreover, it seems that addicts tend to replace foods that are rich in fat and proteins with diet relatively poor in vitamins (20).

In conclusion, our study showed that opium addiction in diabetic patients with CAD may lead to reduction in vitamin A and carbohydrate intake with subsequent ill effect. Besides, any possible change in blood sugar level among addict patients is attributable to their decreased intake of carbohydrates.
